# Supplementary material for: High expression of interleukin-18 receptor alpha correlates with severe respiratory viral disease and defines T cells with reduced cytotoxic signatures
Source: Nat Commun. 2025 Nov 24;16:10344. doi: 10.1038/s41467-025-65262-5 (PMC12644894; doi:10.1038/s41467-025-65262-5)
Supplement: Supplementary file 1 — Supplementary Information [file 41467_2025_65262_MOESM1_ESM.pdf]

SUPPLEMENTARY INFORMATION

High expression of interleukin-18 receptor alpha correlates with severe respiratory viral disease and defines T cells with reduced cytotoxic signatures

Aira F Cabug[1], Jeremy Chase Crawford[2,3], Hayley A McQuilten[1], Isabelle J Foo[1], Lilith F Allen[1], Deborah Gebregzabher[1], Robert C Mettelman[2], Tanya Novak[4], Janet Chou[5], Louise C Rowntree[1], Ruth R Hagen[1], Abby J Thomson[1], Genevieve E Martin[1,6], Brad Gilbertson[1], Michael NT Souter[1], Fiona James[6], Emma Goodall[6], Simone Rizzetto[7], Tim Flerlage[8], Xiaoxiao Jia[1], Lee-Ann Van de Velde[2], So Young Chang[1], Fabio Luciani[7], Ryan S Thwaites[9], Jason A. Trubiano[10-13], Tom C Kotsimbos[14,15], Allen C Cheng[16,17], Adrienne G Randolph[4,18], Paul G Thomas[2,18], Jianqing Xu[19], Zhongfang Wang[1,20], Thi H O Nguyen[1#], Brendon Y Chua[1#], Lukasz Kedzierski[1#] and Katherine Kedzierska[1,18#]

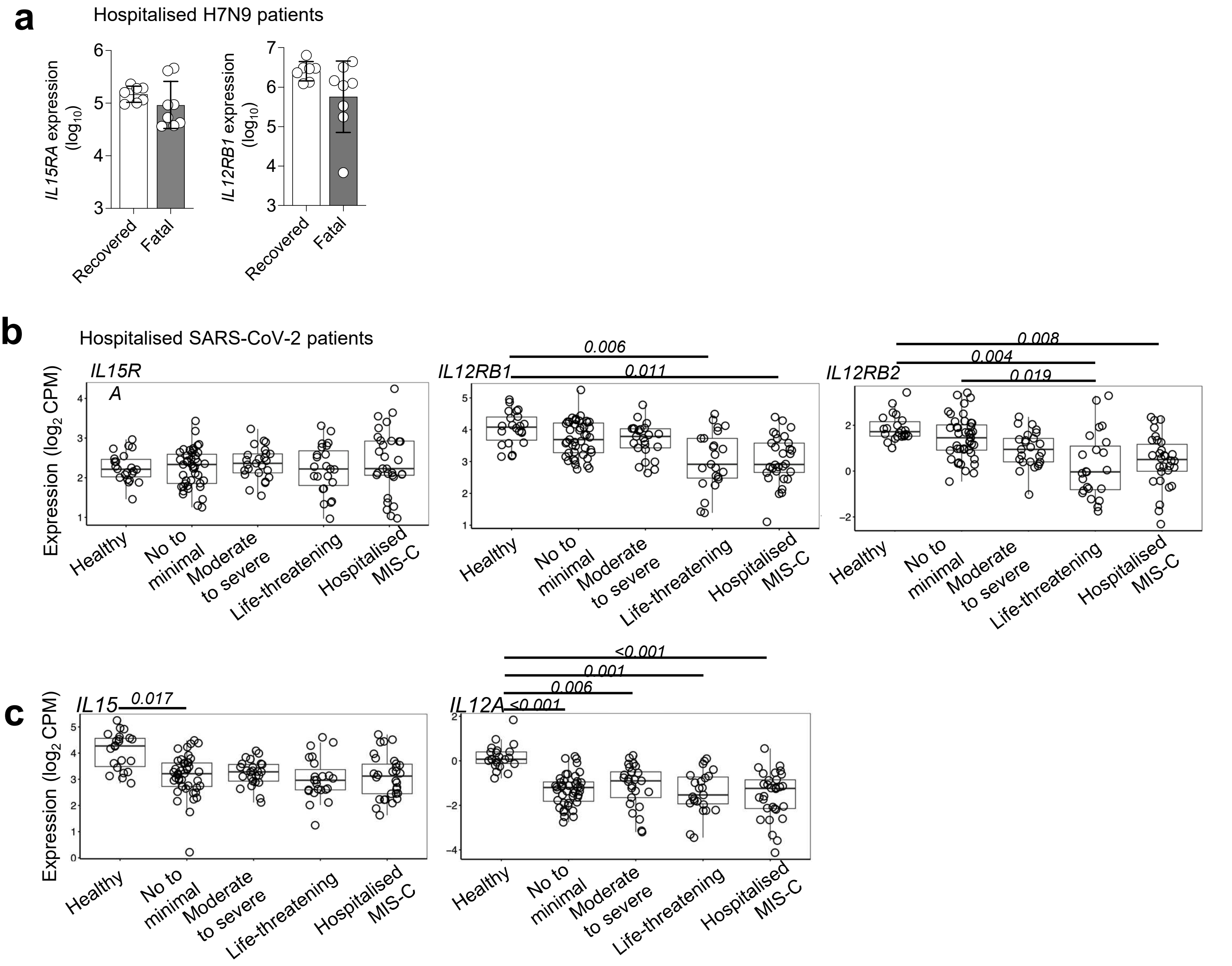

Supplementary Figure 1. Transcript expression levels of *IL15RA* and *IL12RB1* in life-threatening H7N9 and COVID-19. (a) *IL15RA* and *IL12RB1* transcript expression levels in A/H7N9 patients from fatal and recovered groups (n=4 per group, early and late timepoints, mean±SD, two-tailed Welch's t-test). **(b)** healthy individuals and patients with SARS-CoV-2 infection across disease severity. **(c)** IL15 and IL12A transcript levels in COVID-19. Boxplots show transcriptional expression of genes of interest as a function of disease severity. P-values were obtained from a model that controlled for the effects of days since symptoms onset, sex, whether a patient was previously healthy, steroid administration prior to sampling, bacterial co-infection, age, race, and ethnicity, and were adjusted for multiple comparisons.

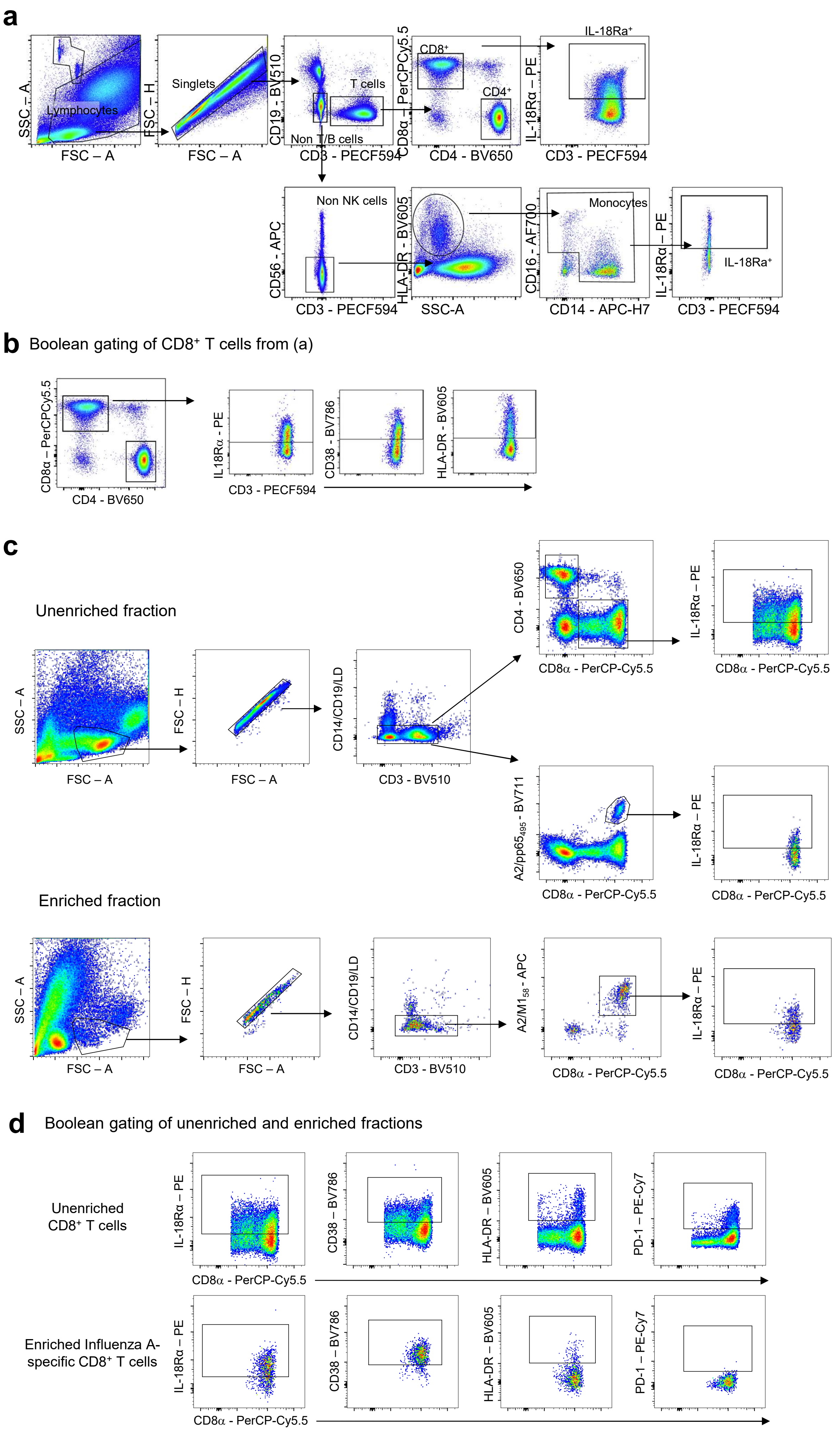

a
Lymphocytes
SSC – A
FSC – A
Singlets
FSC – H
FSC – A
CD19 - BV510
T cells
Non T/B cells
CD3 - PECF594
CD8α – PerCPCy5.5
CD8+
CD4+
CD4 - BV650
IL-18Ra+
IL-18Rα – PE
CD3 - PECF594
Non NK cells
CD56 - APC
CD3 - PECF594
HLA-DR - BV605
SSC-A
Monocytes
CD16 - AF700
CD14 - APC-H7
IL-18Rα – PE
IL-18Ra+
CD3 - PECF594
b Boolean gating of CD8+ T cells from (a)
CD8α – PerCPCy5.5
CD4 - BV650
IL18Rα - PE
CD38 - BV786
HLA-DR - BV605
CD3 - PECF594
c
Unenriched fraction
SSC – A
FSC – A
FSC – H
FSC – A
CD14/CD19/LD
CD3 - BV510
CD4 - BV650
CD8α - PerCP-Cy5.5
IL-18Rα – PE
CD8α - PerCP-Cy5.5
A2/pp65495 - BV711
CD8α - PerCP-Cy5.5
IL-18Rα – PE
CD8α - PerCP-Cy5.5
Enriched fraction
SSC – A
FSC – A
FSC – H
FSC – A
CD14/CD19/LD
CD3 - BV510
A2/M158 - APC
CD8α - PerCP-Cy5.5
IL-18Rα – PE
CD8α - PerCP-Cy5.5
d Boolean gating of unenriched and enriched fractions
Unenriched
CD8+ T cells
IL-18Rα – PE
CD38 – BV786
HLA-DR – BV605
PD-1 – PE-Cy7
CD8α - PerCP-Cy5.5
Enriched Influenza A-specific CD8+ T cells
IL-18Rα – PE
CD38 – BV786
HLA-DR – BV605
PD-1 – PE-Cy7
CD8α - PerCP-Cy5.5

Supplementary Figure 2. Gating strategies for surface IL-18Rα expression in Melbourne patient cohort. (a) Representative gating strategy for IL-18Rα expression in immune cell subsets from human whole blood samples. (b) Boolean gating strategy of IL-18Rα, CD38 and HLA-DR on $CD8^+$ T cells from (a). (c) Representative gating strategy for IL-18Rα expression on unenriched $CD8^+$ T cells, unenriched CMV-specific $CD8^+$ T cells and enriched influenza-specific $CD8^+$ T cells. (d) Boolean gating strategy of IL-18Rα, CD38, HLA-DR and PD-1 from unenriched and enriched $CD8^+$ T cell populations from (c).

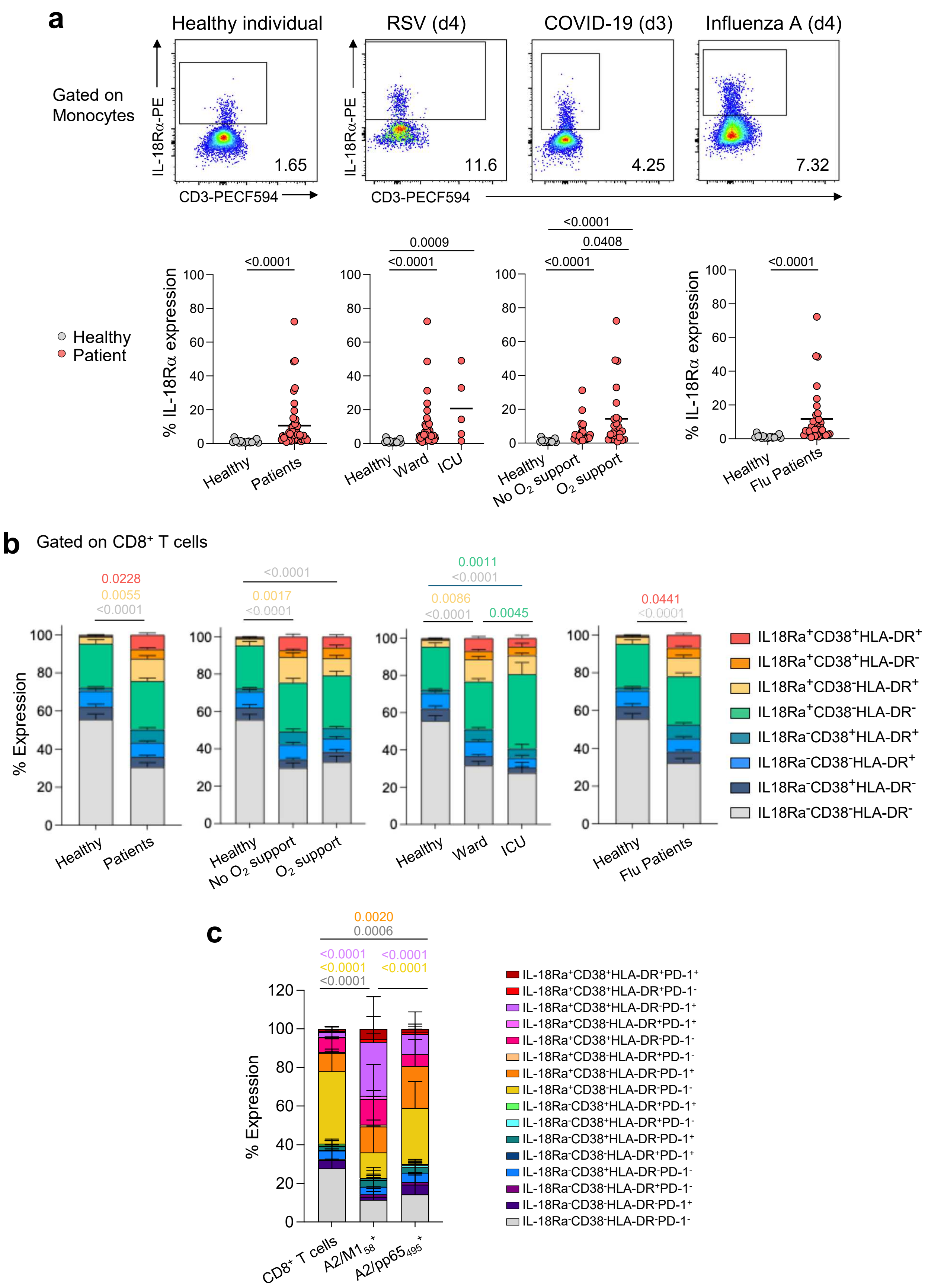
a
Healthy individual
RSV (d4)
COVID-19 (d3)
Influenza A (d4)
Gated on Monocytes
IL-18Rα-PE
CD3-PECF594
1.65
11.6
4.25
7.32
Healthy
Patient
% IL-18Rα expression
<0.0001
0.0009
<0.0001
<0.0001
0.0408
<0.0001
<0.0001
Healthy
Patients
Ward
ICU
No O2 support
O2 support
Flu Patients
b
Gated on CD8+ T cells
% Expression
0.0228
0.0055
<0.0001
0.0017
0.0011
0.0086
0.0045
0.0441
IL18Ra+CD38+HLA-DR+
IL18Ra+CD38+HLA-DR-
IL18Ra+CD38-HLA-DR+
IL18Ra+CD38-HLA-DR-
IL18Ra-CD38+HLA-DR+
IL18Ra-CD38-HLA-DR+
IL18Ra-CD38+HLA-DR-
IL18Ra-CD38-HLA-DR-
c
0.0020
0.0006
<0.0001
% Expression
CD8+ T cells
A2/M1 58+
A2/pp65 495+
IL-18Ra+CD38+HLA-DR+PD-1+
IL-18Ra+CD38+HLA-DR+PD-1-
IL-18Ra+CD38+HLA-DR-PD-1+
IL-18Ra+CD38-HLA-DR+PD-1+
IL-18Ra+CD38+HLA-DR-PD-1-
IL-18Ra+CD38-HLA-DR+PD-1-
IL-18Ra+CD38-HLA-DR-PD-1+
IL-18Ra+CD38-HLA-DR-PD-1-
IL-18Ra-CD38+HLA-DR+PD-1+
IL-18Ra-CD38+HLA-DR+PD-1-
IL-18Ra-CD38+HLA-DR-PD-1+
IL-18Ra-CD38-HLA-DR+PD-1+
IL-18Ra-CD38+HLA-DR-PD-1-
IL-18Ra-CD38-HLA-DR+PD-1-
IL-18Ra-CD38-HLA-DR-PD-1+
IL-18Ra-CD38-HLA-DR-PD-1-

Supplementary Figure 3. IL-18Rα expression on human monocytes and co-expression of activation markers. (a) Representative FACS plots of surface IL-18Rα expression on monocytes in a healthy individual and in hospitalized RSV, COVID-19 and influenza A patients are shown; d: days post disease onset. Graphed IL-18Rα expression in healthy individuals (n=17) and patients hospitalized with influenza A (n=37), influenza B (n=1), RSV (n=4) and COVID-19 (n=2) at all visit (V) time points, grouped by ICU and ward or oxygen support is shown. IL-18Rα expression in healthy individuals versus influenza A patients is shown at hospital visit 1 (V1). Bars indicate median. Statistical significance was analysed by two-tailed Mann-Whitney or Kruskal-Wallis. (b) Co-expression of IL-18Rα, CD38 and HLA-DR on $CD8^+$ T cell population from Melbourne patient cohort as described in (a). (c) Co-expression of IL-18Rα, CD38, HLA-DR and PD-1 on unenriched $CD8^+$ T cells, tetramer-enriched influenza-specific $A2/M1_{58}{}^+CD8^+$ T cells and unenriched CMV-specific $A2/pp65_{495}{}^+CD8^+$ T cells in influenza A patients. (b, c) Stacked columns indicate mean+SD. Statistical significance was analysed by a two-tailed Tukey's multiple comparisons test.

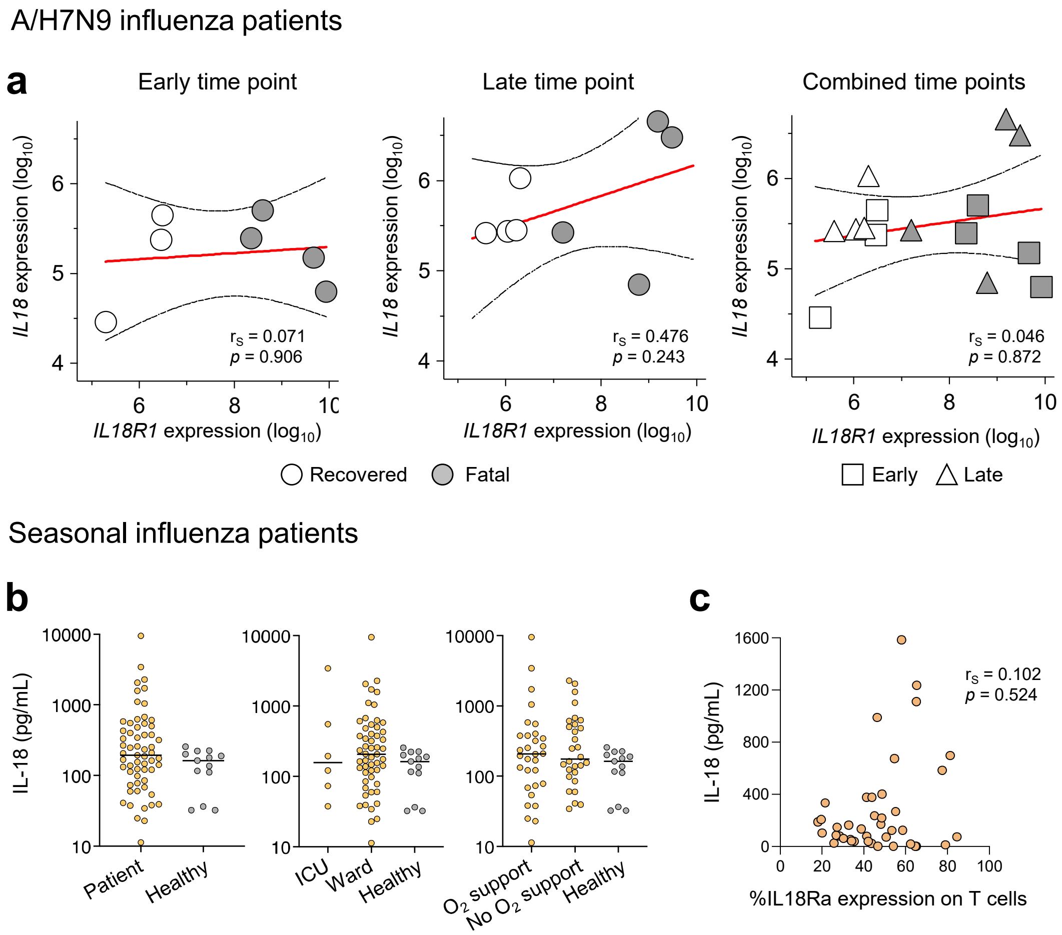

Supplementary Figure 4. Lack of correlation between IL-18Rα and IL-18 cytokine levels in patients infected with influenza viruses. (a) *IL18R1* and *IL18* expression in A/H7N9 patients with fatal and recovery disease outcomes at both early and late time points after hospital admission was assessed using Spearman's rank correlation (r_s). (b) Plasma IL-18 cytokine levels in hospitalized influenza A-infected patients (n=61) and healthy controls (n=13). Patients were grouped according to ICU (n=6) or ward (n=55) compared to healthy controls (n=13) or whether oxygen support was required (O2: n=31, No O_2: n=30). Bars show median (two-tailed Mann-Whitney). (c) IL-18 cytokine levels versus IL-18Rα cell surface expression on $CD3^+$ T cells (Spearman's rank correlation). Only significant differences are shown.

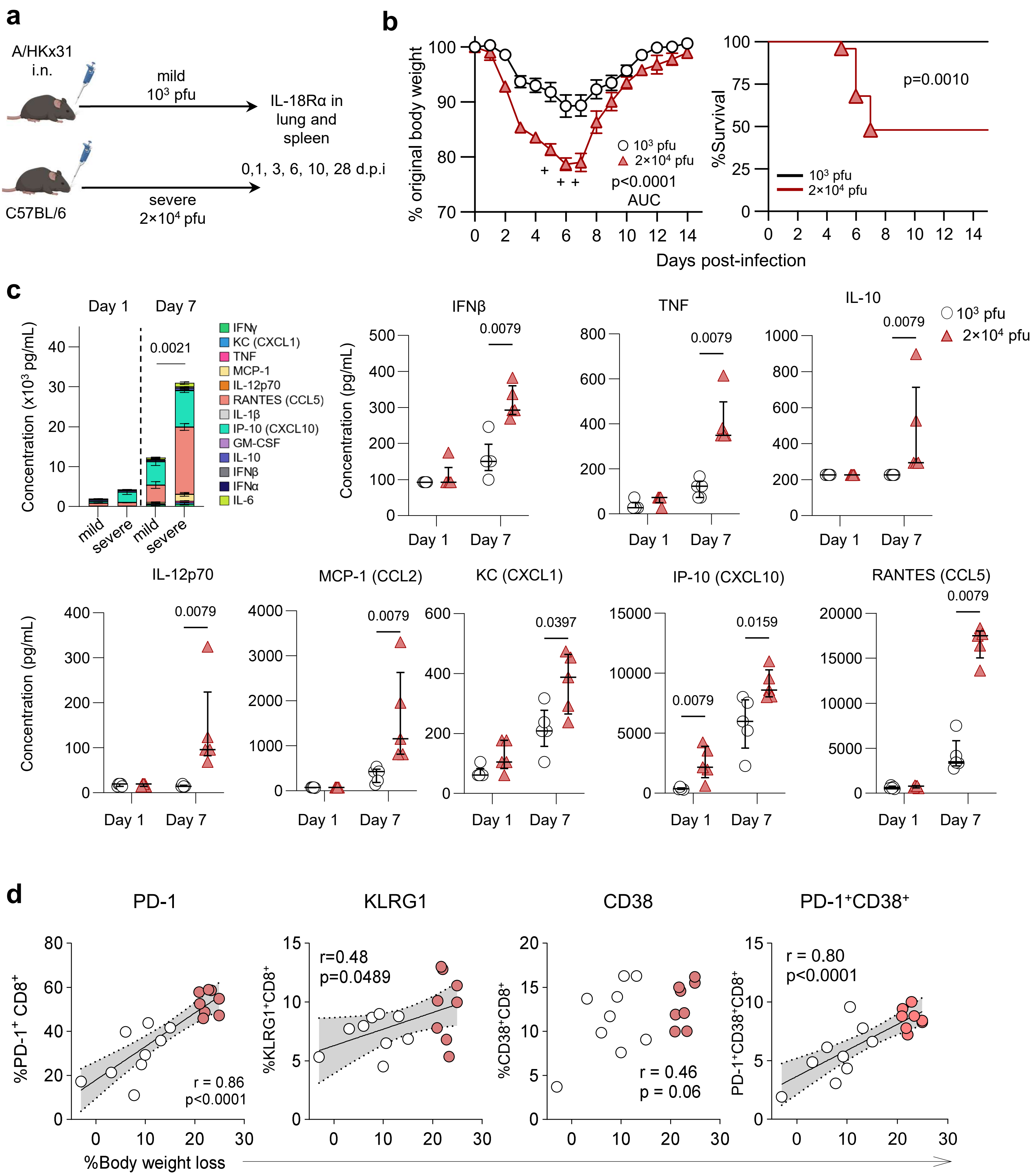
a
A/HKx31
i.n.
mild
10³ pfu
IL-18Rα in
lung and
spleen
0,1, 3, 6, 10, 28 d.p.i
C57BL/6
severe
2×10⁴ pfu
b
% original body weight
10³ pfu
2×10⁴ pfu
p<0.0001
AUC
%Survival
p=0.0010
Days post-infection
c
Day 1
Day 7
Concentration (x10³ pg/mL)
0.0021
mild
severe
IFNγ
KC (CXCL1)
TNF
MCP-1
IL-12p70
RANTES (CCL5)
IL-1β
IP-10 (CXCL10)
GM-CSF
IL-10
IFNβ
IFNα
IL-6
IFNβ
TNF
IL-10
Concentration (pg/mL)
0.0079
IL-12p70
MCP-1 (CCL2)
KC (CXCL1)
IP-10 (CXCL10)
RANTES (CCL5)
0.0397
0.0159
d
PD-1
KLRG1
CD38
PD-1⁺CD38⁺
%PD-1⁺ CD8⁺
%KLRG1⁺CD8⁺
%CD38⁺CD8⁺
PD-1⁺CD38⁺CD8⁺
r = 0.86
p<0.0001
r=0.48
p=0.0489
r = 0.46
p = 0.06
r = 0.80
p<0.0001
%Body weight loss

Supplementary Figure 5. Severe influenza virus infection is characterised by hypercytokinaemia and elevated lung damage. (a) C57BL/6 mice were challenged i.n. with a low (10^3 pfu) and high (2×10^4 pfu) dose of A/HKx31 to model mild and severe influenza disease, respectively. Analyses were performed at multiple timepoints following infection. Created in BioRender. Cabug, A. (2025) https://BioRender.com/q5po0oj. (b) Body weight loss and survival were monitored daily for 14 days following IAV infection. The overall difference in weight loss between mild (n=25) and severe (n=15) groups was compared using area under the curve (AUC) analysis. Survival was compared using a Log-Rank (Mantel-Cox) test. (c) Cytokine and chemokine levels were measured from the lung homogenates of IAV-infected mice (n_{mild}=5, n_{severe}=5) on days 1 and 7 post-infection. Overall cytokine composition of the lungs of mild and severe groups are shown. Bars are mean ± SEM. Cytokines with statistically significant differences between mild and severe groups were determined using a two-tailed Mann-Whitney U-test. Bars are median and interquartile range (IQR). (d) Correlations between PD-1, CD38, KLRG1 and PD-1/CD38 on $CD8^+$ T cells with the body weight loss.

a

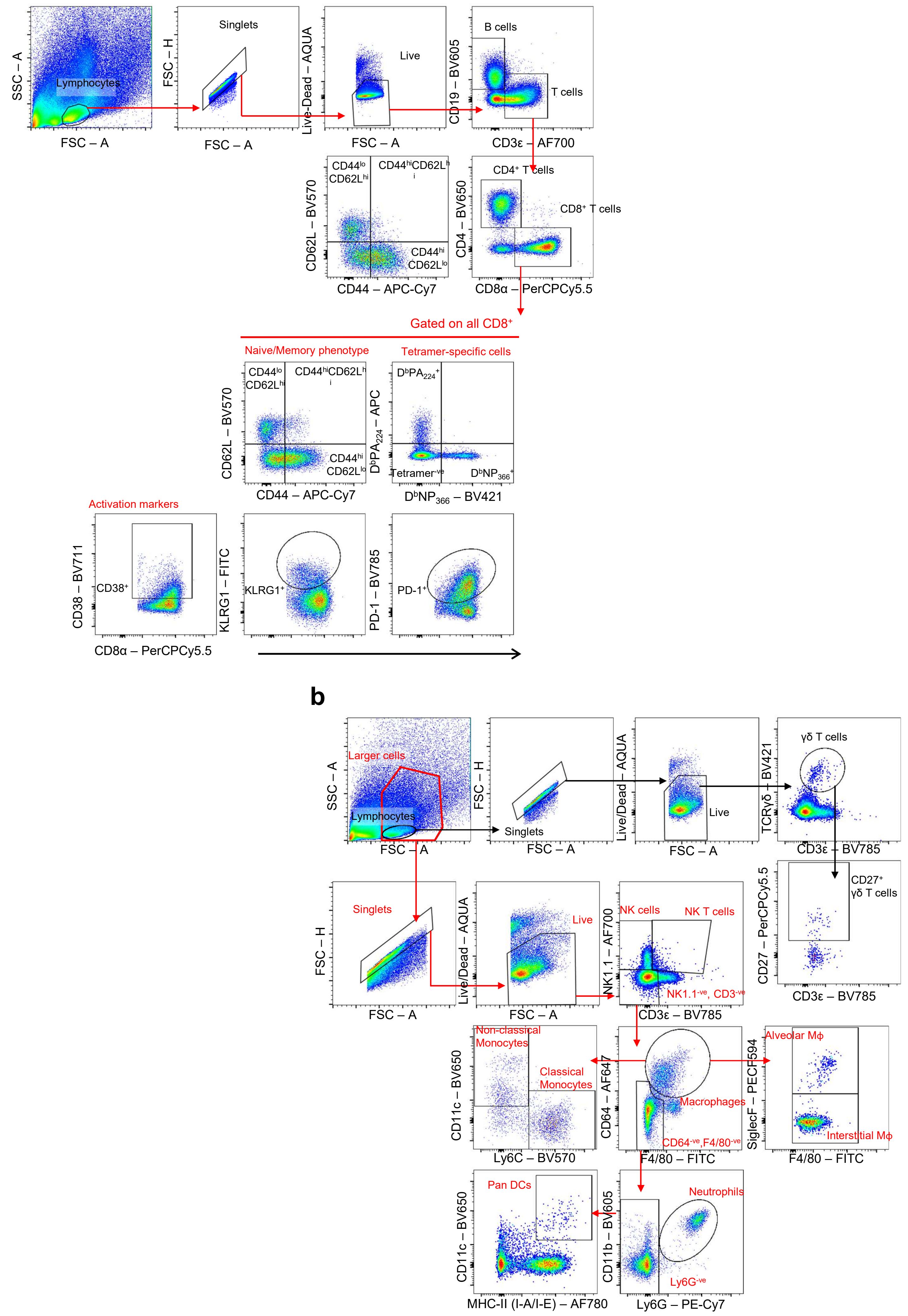

Lymphocytes
SSC – A
FSC – A
Singlets
FSC – H
FSC – A
Live
Live-Dead – AQUA
FSC – A
B cells
T cells
CD19 – BV605
CD3ε – AF700
CD44lo CD62Lhi
CD44hiCD62Lhi
CD44hi CD62Llo
CD62L – BV570
CD44 – APC-Cy7
CD4+ T cells
CD8+ T cells
CD4 – BV650
CD8α – PerCPCy5.5
Gated on all CD8+
Naive/Memory phenotype
Tetramer-specific cells
CD44lo CD62Lhi
CD44hiCD62Lhi
CD44hi CD62Llo
CD62L – BV570
CD44 – APC-Cy7
DbPA224+
Tetramer-ve
DbNP366+
DbPA224 – APC
DbNP366 – BV421
Activation markers
CD38+
CD38 – BV711
CD8α – PerCPCy5.5
KLRG1+
KLRG1 – FITC
PD-1+
PD-1 – BV785
b
Larger cells
Lymphocytes
SSC – A
FSC – A
Singlets
FSC – H
FSC – A
Live
Live/Dead – AQUA
FSC – A
γδ T cells
TCRγδ – BV421
CD3ε – BV785
CD27+ γδ T cells
CD27 – PerCPCy5.5
CD3ε – BV785
Singlets
FSC – H
FSC – A
Live
Live/Dead – AQUA
FSC – A
NK cells
NK T cells
NK1.1-ve, CD3-ve
NK1.1 – AF700
CD3ε – BV785
Non-classical Monocytes
Classical Monocytes
CD11c – BV650
Ly6C – BV570
Macrophages
CD64-ve,F4/80-ve
CD64 – AF647
F4/80 – FITC
Alveolar Mϕ
Interstitial Mϕ
SiglecF – PECF594
F4/80 – FITC
Pan DCs
CD11c – BV650
MHC-II (I-A/I-E) – AF780
Neutrophils
Ly6G-ve
CD11b – BV605
Ly6G – PE-Cy7

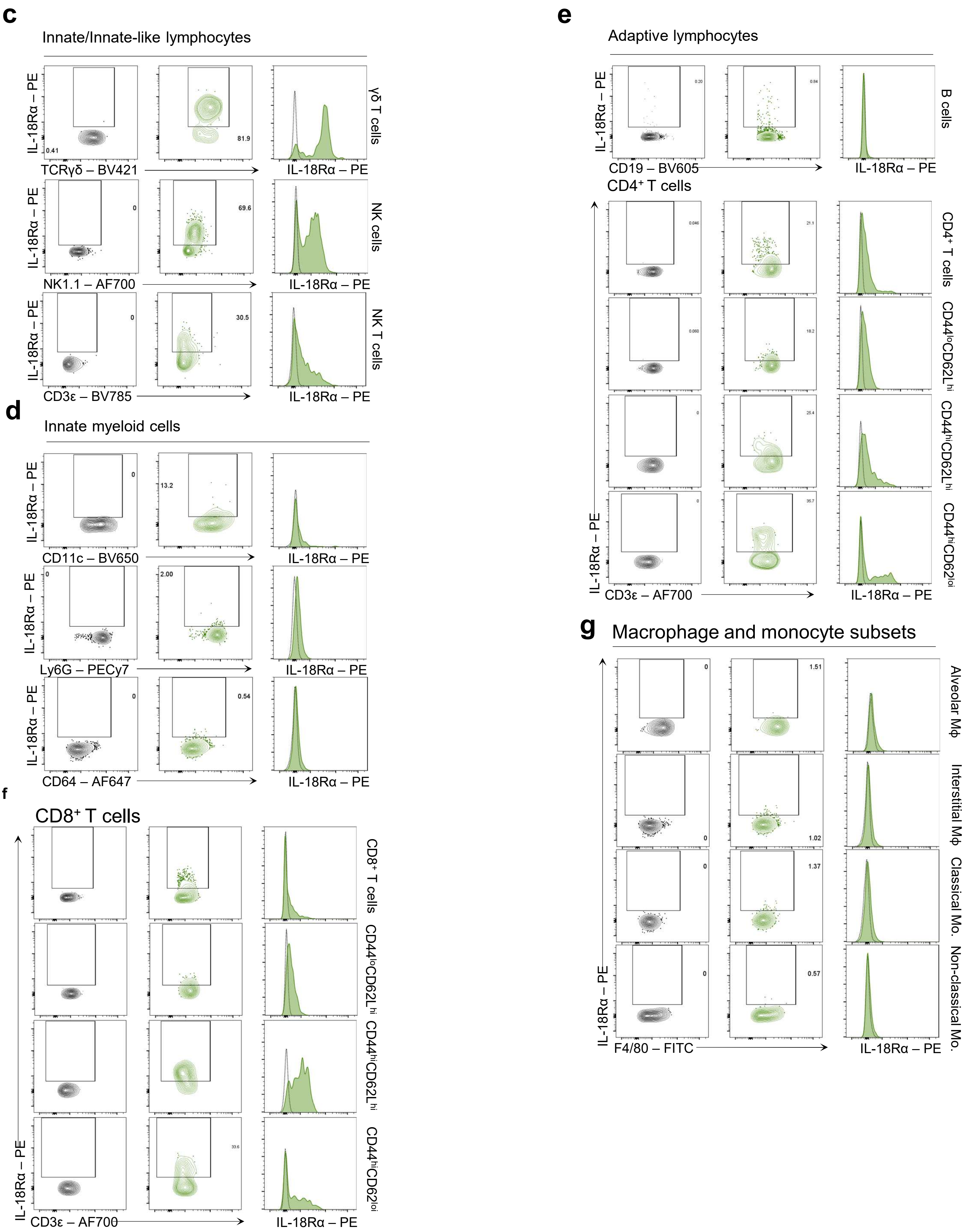

Supplementary Figure 6. IL-18Rα expression on mouse immune cell subsets. Representative gating strategy for the identification of (a) adaptive immune cells and (b) innate immune cells referred to in Fig. 4. (c-g) Representative FACS plots and histograms showing steady state expression levels of IL-18Rα on innate and adaptive immune cells. Plots on left (black) represent the corresponding isotype control.

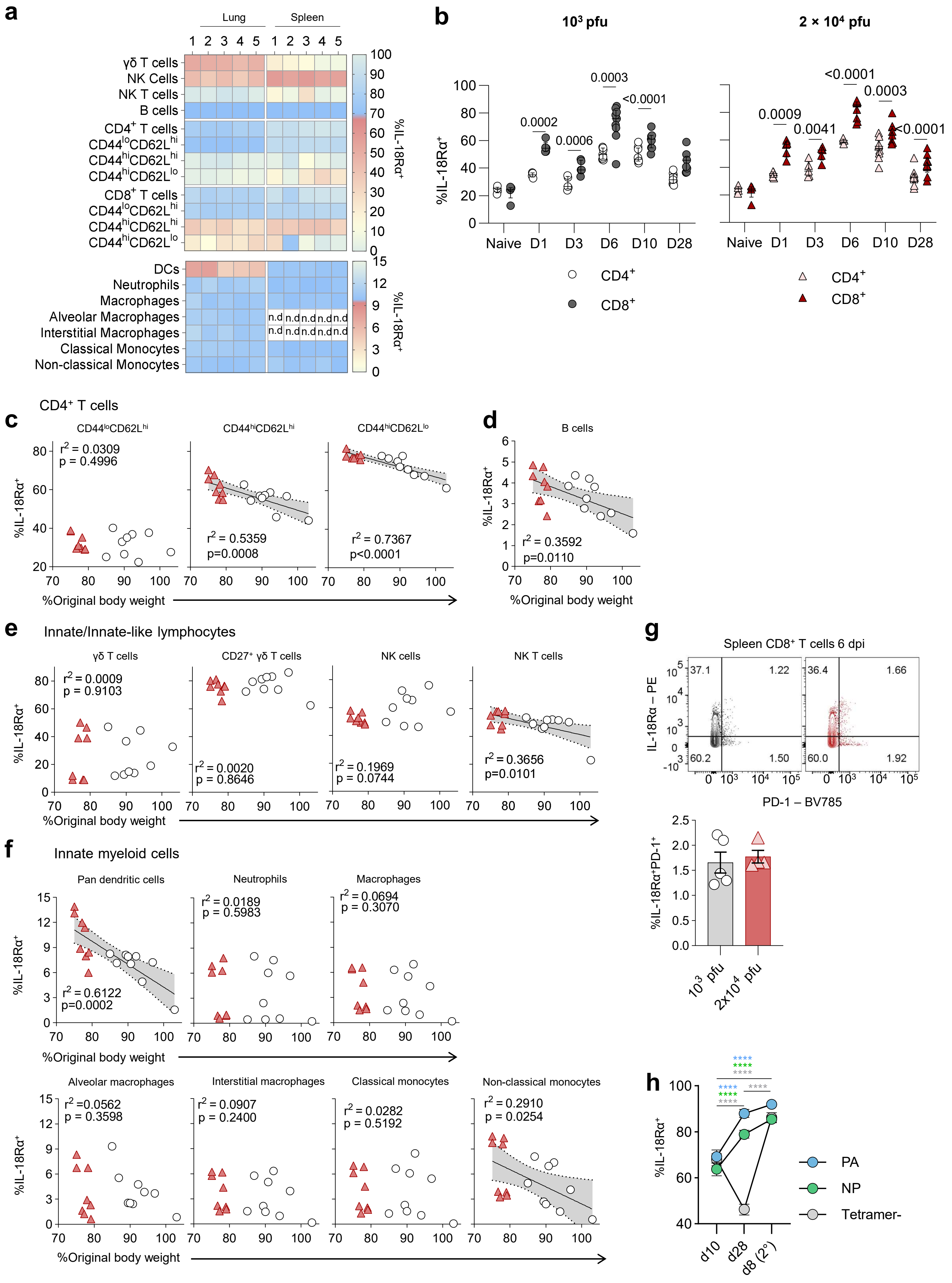

a
Lung
Spleen
γδ T cells
NK Cells
NK T cells
B cells
CD4+ T cells
CD44loCD62Lhi
CD44hiCD62Lhi
CD44hiCD62Llo
CD8+ T cells
CD44loCD62Lhi
CD44hiCD62Lhi
CD44hiCD62Llo
%IL-18Rα+
DCs
Neutrophils
Macrophages
Alveolar Macrophages
Interstitial Macrophages
Classical Monocytes
Non-classical Monocytes
n.d
b
10³ pfu
2 × 10⁴ pfu
%IL-18Rα+
Naive D1 D3 D6 D10 D28
0.0002
0.0006
0.0003
<0.0001
0.0009
0.0041
<0.0001
0.0003
<0.0001
CD4+
CD8+
CD4+ T cells
c
CD44loCD62Lhi
CD44hiCD62Lhi
CD44hiCD62Llo
r² = 0.0309
p = 0.4996
r² = 0.5359
p=0.0008
r² = 0.7367
p<0.0001
%Original body weight
d
B cells
r² = 0.3592
p=0.0110
e
Innate/Innate-like lymphocytes
γδ T cells
CD27+ γδ T cells
NK cells
NK T cells
r² = 0.0009
p = 0.9103
r² = 0.0020
p = 0.8646
r² = 0.1969
p = 0.0744
r² = 0.3656
p=0.0101
f
Innate myeloid cells
Pan dendritic cells
Neutrophils
Macrophages
r² = 0.6122
p=0.0002
r² = 0.0189
p = 0.5983
r² = 0.0694
p = 0.3070
Alveolar macrophages
Interstitial macrophages
Classical monocytes
Non-classical monocytes
r² =0.0562
p = 0.3598
r² = 0.0907
p = 0.2400
r² = 0.0282
p = 0.5192
r² = 0.2910
p = 0.0254
g
Spleen CD8+ T cells 6 dpi
IL-18Rα – PE
PD-1 – BV785
37.1
1.22
60.2
1.50
36.4
1.66
60.0
1.92
%IL-18Rα+PD-1+
10³ pfu
2x10⁴ pfu
h
%IL-18Rα+
d10
d28
d8 (2°)
PA
NP
Tetramer-
****

Supplementary Figure 7. Correlations between IL-18Rα expression levels on immune cell subsets and body weight loss. (a) Heatmap showing the mean expression (%IL-18Rα$^+$) on innate and adaptive immune cell types in the lung and spleen in naïve mice. (b) Graphs comparing the expression levels of IL-18Rα on CD4$^+$ and CD8$^+$ T-cells at various timepoints following either low-dose (10^3 pfu, left) or high-dose ($2x10^4$ pfu, right) A/HKx31infection. (c-f) Correlation between body weight loss and IL-18Rα expression levels on (C) CD4$^+$ T-cell subsets based on CD44 and CD62L expression, (d) on B-cells, (e) on innate-like lymphocytes and (f) on innate myeloid cells. Correlation between body weight loss and IL-18Rα expression on immune cells was assessed using Pearson's correlation (r^2). Shaded bars represent the 95% confidence interval. (g) Representative FACS plots showing double-positive IL-18Rα$^+$ PD-1$^+$ CD8$^+$ T-cells in the spleen at 6 dpi (top). Bar graph below shows comparison of IL-18Rα$^+$ PD-1$^+$ CD8$^+$ T-cells in low and high dose A/HKx31 infection. (h) Graphs showing the change in IL-18Rα$^+$ expression on tetramer$^-$, NP- and PA-specific CD8$^+$ T-cells at 10 and 28 dpi, and 8 days post-secondary infection. IL-18Rα expression on tetramer$^-$, NP- and PA-specific CD8$^+$ T-cells at different timepoints was compared using a two-way ANOVA with Tukey's correction for multiple comparisons. Bars in (b) show median and interquartile range (IQR) and bars in (G-H) show mean ± SEM. **P<0.01;***P<0.001;****P<0.0001; n.s = not significant.

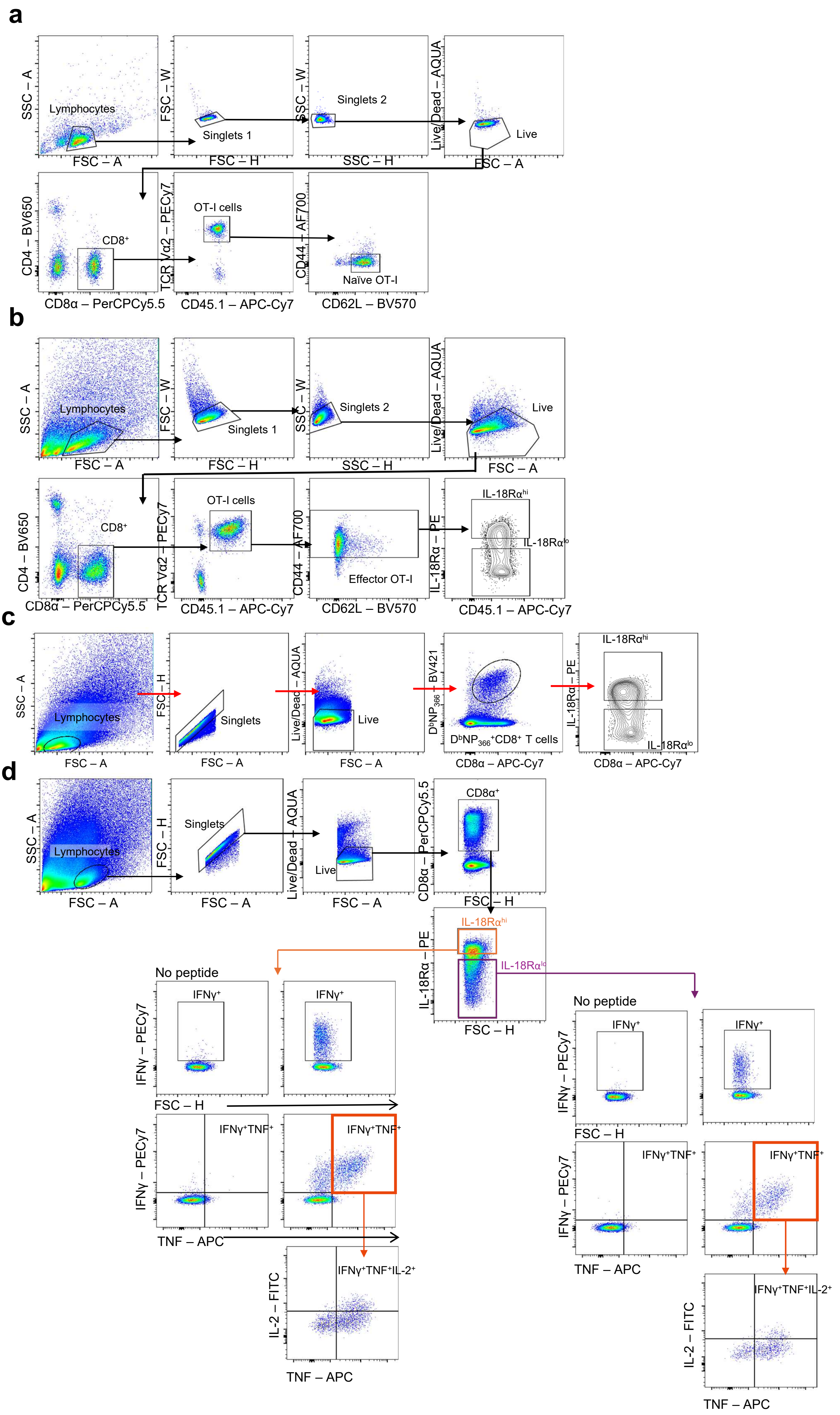
a
SSC – A
Lymphocytes
FSC – A
FSC – W
Singlets 1
FSC – H
SSC – W
Singlets 2
SSC – H
Live/Dead – AQUA
Live
FSC – A
CD4 – BV650
CD8⁺
CD8α – PerCPCy5.5
TCR Vα2 – PECy7
OT-I cells
CD45.1 – APC-Cy7
CD44 – AF700
Naïve OT-I
CD62L – BV570
b
SSC – A
Lymphocytes
FSC – A
FSC – W
Singlets 1
FSC – H
SSC – W
Singlets 2
SSC – H
Live/Dead – AQUA
Live
FSC – A
CD4 – BV650
CD8⁺
CD8α – PerCPCy5.5
TCR Vα2 – PECy7
OT-I cells
CD45.1 – APC-Cy7
CD44 – AF700
Effector OT-I
CD62L – BV570
IL-18Rα – PE
IL-18Rαhi
IL-18Rαlo
CD45.1 – APC-Cy7
c
SSC – A
Lymphocytes
FSC – A
FSC – H
Singlets
FSC – A
Live/Dead – AQUA
Live
FSC – A
DbNP366 – BV421
DbNP366⁺CD8⁺ T cells
CD8α – APC-Cy7
IL-18Rα – PE
IL-18Rαhi
IL-18Rαlo
CD8α – APC-Cy7
d
SSC – A
Lymphocytes
FSC – A
FSC – H
Singlets
FSC – A
Live/Dead – AQUA
Live
FSC – A
CD8α – PerCPCy5.5
CD8α⁺
FSC – H
IL-18Rα – PE
IL-18Rαhi
IL-18Rαlo
FSC – H
No peptide
IFNγ – PECy7
IFNγ⁺
FSC – H
IFNγ⁺TNF⁺
TNF – APC
IL-2 – FITC
IFNγ⁺TNF⁺IL-2⁺
TNF – APC

Supplementary Figure 8. Representative FACS plots of mouse experiments. (a) Representative gating strategy for the isolation of naïve CD45.1$^+$ OT-I T-cells from pooled lymph nodes via FACS sorting, related to Fig. 4. (b) Representative gating strategy for the isolation of activated IL-18Rα^{hi} and IL-18Rα^{lo} OT-I T-cells at 6 dpi from the lung, related to Fig. 4. (c) Representative gating strategy showing the identification of tetramer-specific IL-18Rα^{hi} and IL-18Rα^{lo}CD8$^+$ T-cells, related to Fig. 5. (d) Representative gating strategy for the identification of cytokine-producing CD8$^+$ IL-18Rα^{hi} and IL-18Rα^{lo}CD8$^+$ T-cells, related to Fig. 6.

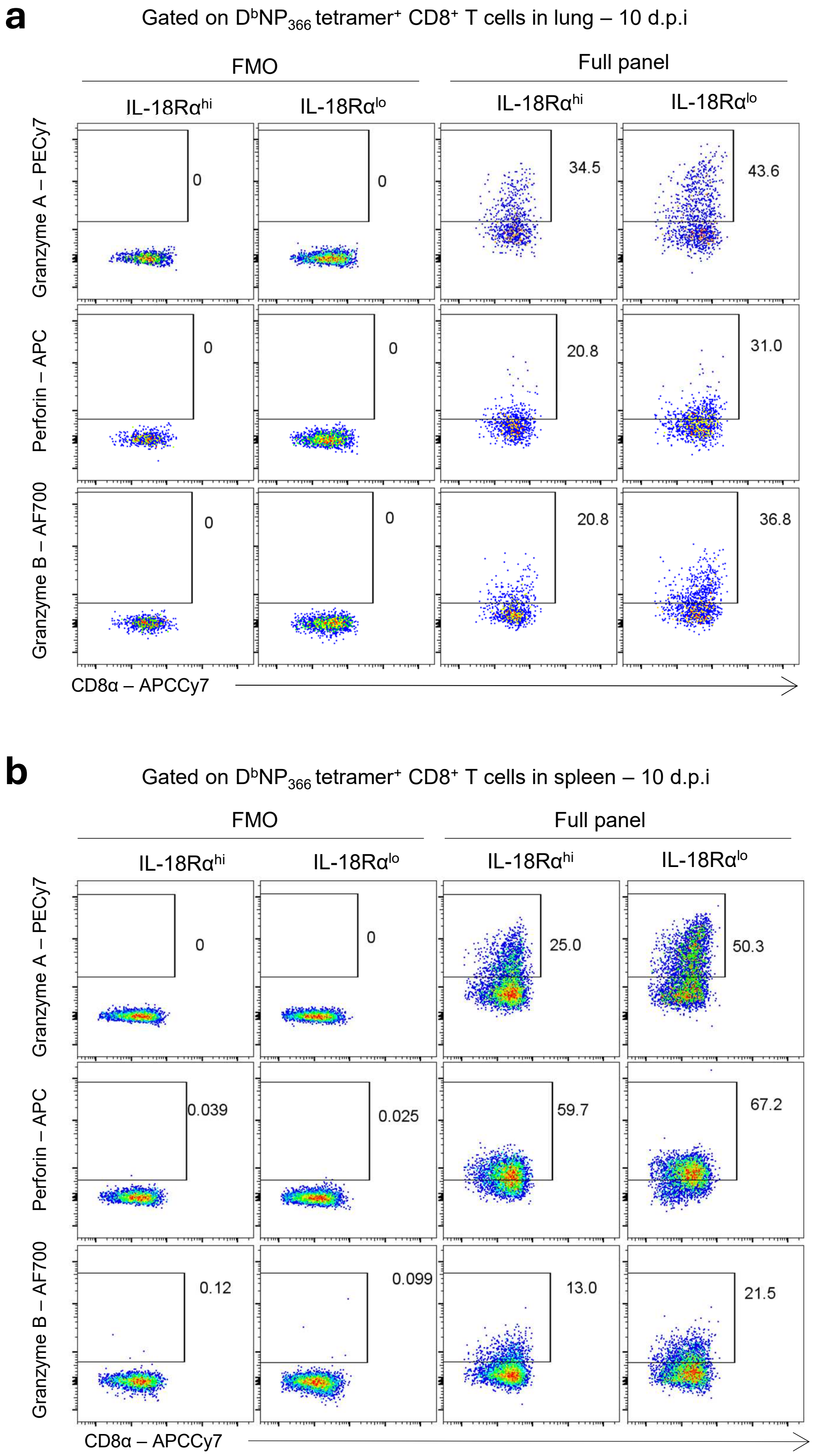

Supplementary Figure 9. Representative FACS plots of FMO controls for granzymes and perforin. Representative FACS plot showing fluorescence minus one (FMO) controls for Granzyme A (top), Perforin (middle), and Granzyme B (bottom) in the (a) lung and (b) spleen. Related to Fig. 6.

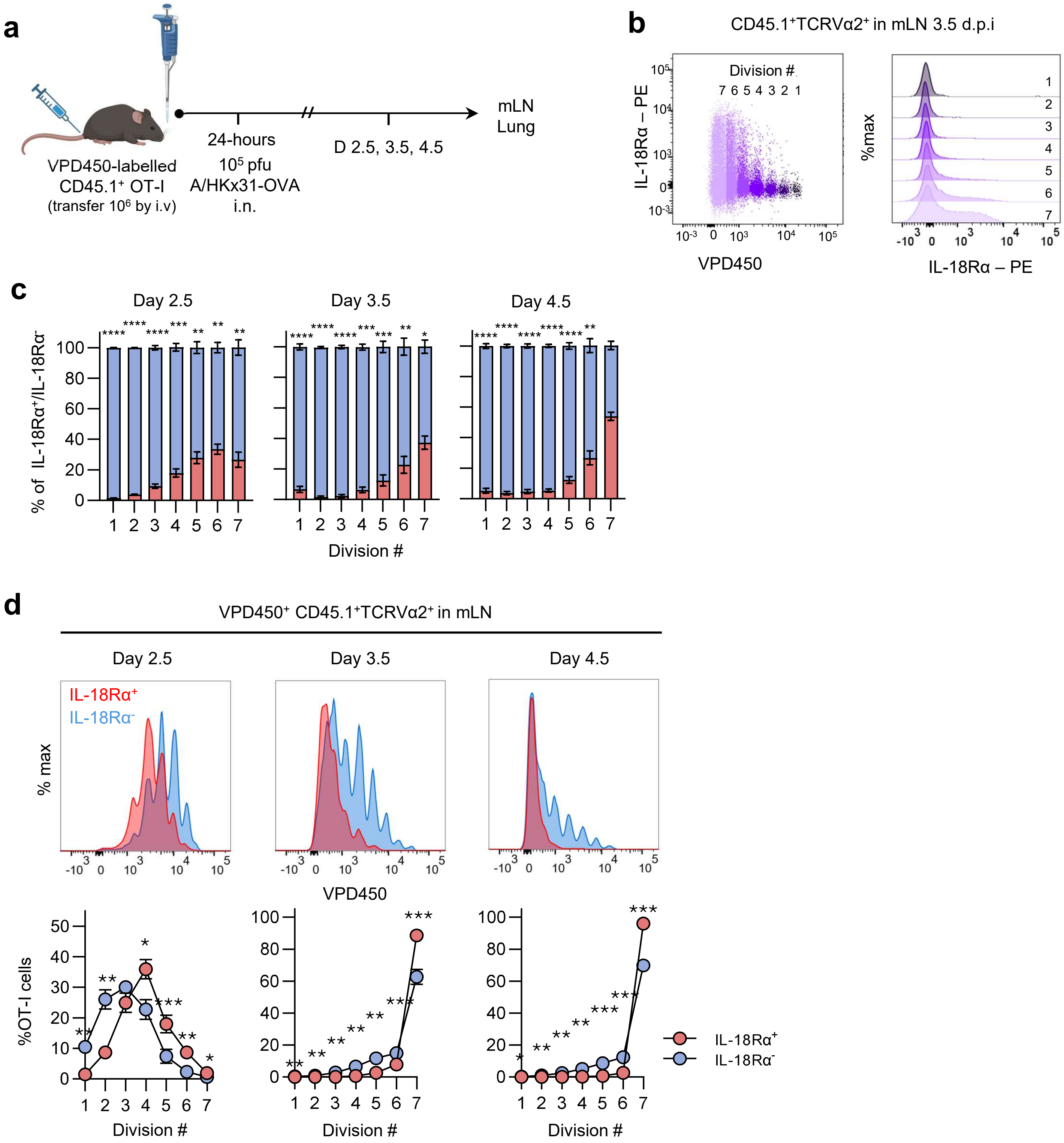
a
VPD450-labelled
CD45.1⁺ OT-I
(transfer 10⁶ by i.v)
24-hours
10⁵ pfu
A/HKx31-OVA
i.n.
D 2.5, 3.5, 4.5
mLN
Lung
b
CD45.1⁺TCRVα2⁺ in mLN 3.5 d.p.i
Division #
7 6 5 4 3 2 1
IL-18Rα – PE
VPD450
%max
IL-18Rα – PE
c
Day 2.5
Day 3.5
Day 4.5
% of IL-18Rα⁺/IL-18Rα⁻
Division #
d
VPD450⁺ CD45.1⁺TCRVα2⁺ in mLN
Day 2.5
Day 3.5
Day 4.5
IL-18Rα⁺
IL-18Rα⁻
% max
VPD450
%OT-I cells
Division #
IL-18Rα⁺
IL-18Rα⁻

Supplementary Figure 10. CD8$^+$ T-cells expressing the IL-18 receptor proliferate efficiently *in vivo* following IAV infection. (a) 10^6 naïve CD45.1$^+$ OT-I cells were labelled with VPD450 and adoptively transferred into C57BL/6 mice. Mice were infected with 10^5 pfu A/HKx31-OVA 24-hours post-transfer and analyses performed in the draining mediastinal lymph node (mLN) and lung. (b) Representative FACS plot and histogram showing IL-18Rα upregulation on OT-I cells with successive rounds of division. (c) Frequency of IL-18Rα$^+$ and IL-18Rα$^-$ OT-I cells in each division stage in the mLN 3.5 dpi. (d) OT-I cells in each division number were analysed for expression of IL-18Rα and frequencies of IL-18Rα$^+$ and IL-18Rα$^-$ cells in the mLN were compared on 2.5, 3.5 and 4.5 dpi. Populations were compared via a paired t-test where *P<0.05, **P<0.01, ***P<0.001.

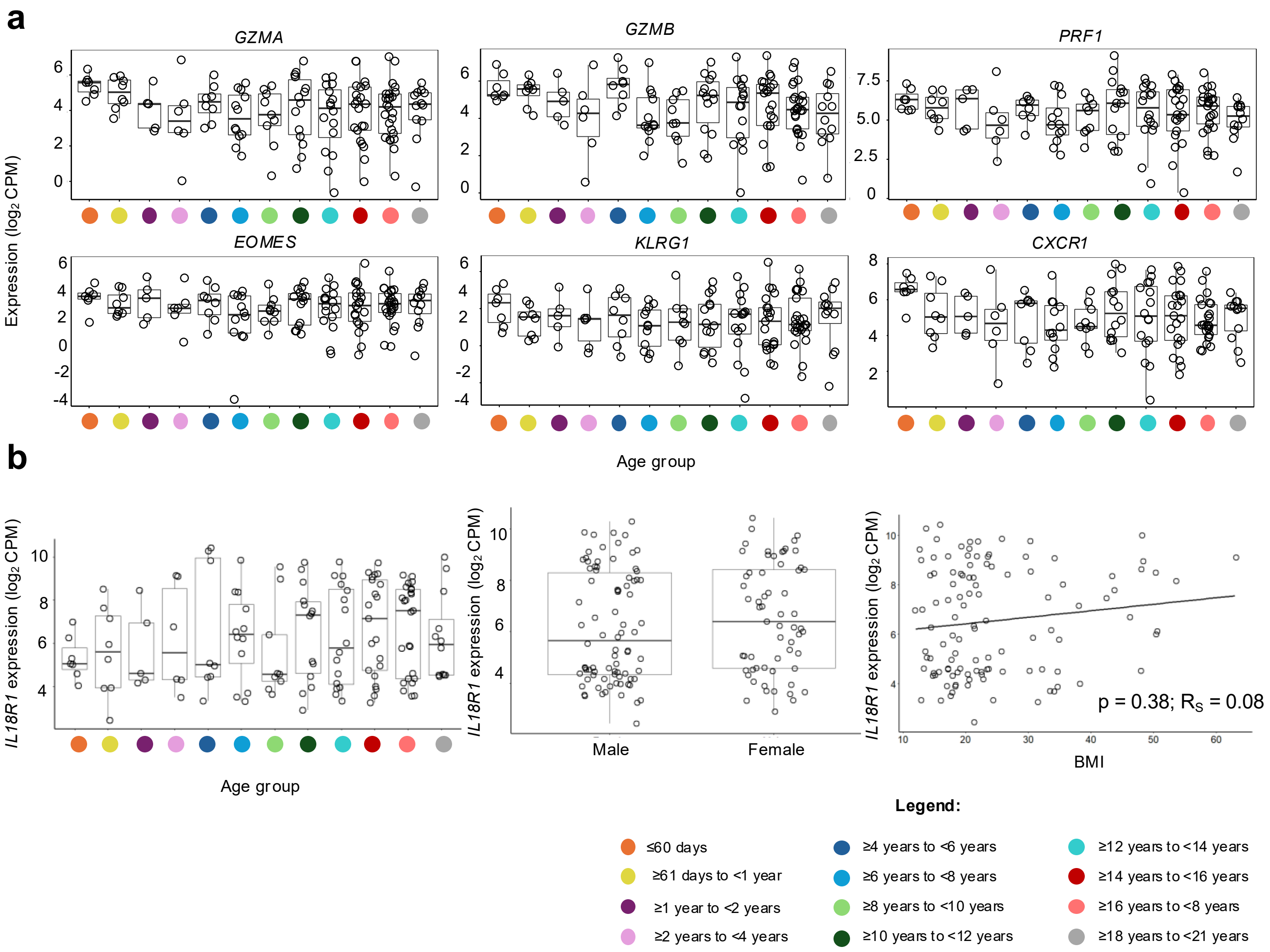

Supplementary Fig. 11. *GZMA*, *GZMB*, *PRF1*, *EOMES*, *KLRG1*, *CXCR1* and IL-18R1 expression levels stratified according to age ranges. (a) Boxplots showing transcriptional expression of genes as a function of age category. (b) *IL18R1* expression did not vary significantly as a function of age category, sex, or BMI (R_S: Spearman's rank correlation).

Supplementary Table 1. Demographics of the Melbourne patient cohort of acute respiratory disease and healthy individuals.

**Cohort Summary**	**Healthy**	**Patients**
Number of individuals, *n*	17	43
Age, median (range)	22 (19-52)	68 (26-89)
Female, *n* (%)	14 (82%)	20 (47%)
Days post hospital admission, median (range)	-	2 (0-45)
Diagnosis		
Influenza A	-	37 (86%)
Influenza B	-	1 (2%)
RSV	-	3 (7%)
SARS-CoV-2	-	2 (5%)
Location in hospital		
Ward	-	41 (95%)
ICU	-	2 (5%)
Level of Oxygen Support		
Oxygen Support	-	20 (47%)
None	-	23 (53%)

Supplementary Table 2. Patient information for IL-18Rα analysis in humans

ID	Gender	Age range	Ethnicity	Number of visits	Days post hospital admission	Diagnosis	Severity	Peptide/HLA-A2-tetramer staining
1	F	55-59	Caucasian	2	1, 32	RSV	Ward	
2	M	70-74	Caucasian	2	0, 33	Influenza A	Ward	
3	M	35-39	Asian	1	2	Influenza A	Ward	
4	F	75-79	Caucasian	2	2, 45 (out of hospital)	Influenza A	Ward	Yes
5	F	25-29	Caucasian	1	1	Influenza A	Ward	
6	M	80-84	Caucasian	1	1	Influenza A	Ward	
7	M	85-89	Caucasian	1	2	Influenza A	Ward	
8	F	65-69	Caucasian	1	4	Influenza A	Ward	
9	M	65-69	Caucasian	1	2	Influenza A	Ward	
10	F	45-49	Caucasian	1	2	Influenza A	Ward	
11	M	50-54	Caucasian	1	2	RSV	Ward	
12	F	75-79	Caucasian	1	1	SARS-CoV-2	Ward	
13	F	40-44	Asian	1	2	RSV	Ward	
14	F	85-89	Caucasian	1	1	Influenza A	Ward	
15	M	60-64	Asian	2	1, 8	Influenza A	Ward	
16	M	70-74	First Nations	1	2	Influenza A	Ward	Yes
17	M	70-74	Caucasian	1	1	Influenza A	Ward	Yes
18	M	50-54	Caucasian	1	1	SARS-CoV-2	Ward	
19	F	65-69	Caucasian	1	3	Influenza A	Ward	
20	M	unk	Caucasian	1	1	Influenza A	Ward	
21	M	85-89	Caucasian	1	1	Influenza A	Ward	
22	M	70-74	Asian	1	5	Influenza B	Ward	
23	F	85-89	Caucasian	2	1, 3	Influenza A	Ward	
24	F	65-69	Caucasian	1	12	Influenza A	Ward, oxygen support	
25	M	70-74	Caucasian	1	3	Influenza A	Ward, oxygen support	
26	F	55-59	Caucasian	1	3	Influenza A	Ward, oxygen support	Yes
27	M	75-79	Caucasian	1	2	Influenza A	Ward, oxygen support	
28	M	80-84	Caucasian	1	1	Influenza A	Ward, oxygen support	Yes
29	M	45-49	Caucasian	1	2	Influenza A	Ward, oxygen support	Yes
30	F	30-34	Caucasian	1	4	Influenza A	Ward, oxygen support	
31	F	45-49	Caucasian	1	4	Influenza A	Ward, oxygen support	
32	M	65-69	Caucasian	1	2	Influenza A	Ward, oxygen support	
33	M	70-74	Caucasian	1	3	Influenza A	Ward, oxygen support	
34	F	55-59	Caucasian	1	1	Influenza A	Ward, oxygen support	
35	F	85-89	Caucasian	1	2	Influenza A	Ward, oxygen support	Yes
36	F	70-74	Asian	1	1	Influenza A	Ward, oxygen support	
37	M	65-69	Caucasian	1	1	Influenza A	Ward, oxygen support	
38	F	70-74	Caucasian	1	1	Influenza A	Ward, oxygen support	
39	M	50-54	Caucasian	1	2	Influenza A	Ward, oxygen support	
40	M	60-64	Caucasian	1	3	Influenza A	Ward, oxygen support	Yes
41	F	60-64	Asian	1	2	Influenza A	Ward, oxygen support	
42	M	50-54	Caucasian	1	3	Influenza A	ICU, Oxygen support	Yes
43	F	40-44	Caucasian	4	1, 5, 8, 15	Influenza A	ICU, Oxygen support	Yes

Supplementary Table 3. Reagents used in the study

**Antibodies and flow cytometry reagents**			
Anti-mouse KLRG1-FITC, Clone 2F1	1:100	Invitrogen	Cat # 11-5893-82 RRID: AB_1311265
Anti-mouse CD8α-PerCP-Cy5.5, Clone 53-6.7	1:200	BD Pharmingen	Cat # 551162 RRID: AB_394081
Anti-mouse CD3-AF700, Clone eBio500A2	1:200	Invitrogen	Cat # 56-0033-82 RRID: AB_837094
Anti-mouse CD44-APC-Cy7, Clone IM7	1:500	BD Pharmingen	Cat # 560568 RRID: AB_1727481
Anti-mouse CD62L-BV570, Clone MEL-14	1:200	BioLegend	Cat # 104433 RRID: AB_10900262
Anti-mouse CD19-BV605, Clone 1D3	1:400	BD Horizon	Cat # 563148 RRID: AB_2732057
Anti-mouse CD4-BV650, Clone RM4.5	1:400	Invitrogen	Cat # 416-0042-82 RRID: AB_2921041
Anti-mouse CD38-BV711, Clone 90/CD38	1:500	BD OptiBuild	Cat # 740697 RRID: AB_2740381
Anti-mouse PD-1 (CD279)-BV785, Clone 29F.A12	1:200	BioLegend	Cat # 135225 RRID: AB_2563680
Anti-mouse IL-18Rα (CD218a)-PE, Clone A17071D	1:200	BioLegend	Cat # 157903 RRID: AB_2860732
Rat Ig2b, κ Isotype Control-PE, Clone RTK4530	1:200	BioLegend	Cat # 400607 RRID: AB_326551
Anti-mouse CD69-PE-Cy7, Clone H1.2F3	1:200	BD Pharmingen	Cat # 561930 RRID: AB_394508
Anti-mouse F4/80-FITC, Clone BM8	1:150	Invitrogen	Cat # 11-4801-82 RRID: AB_2637191
Anti-mouse/rat/human CD27-PerCPCy5.5, Clone LG.3A10	1:400	BioLegend	Cat # 124213 RRID: AB_2073424

Anti-mouse CD64-AF647, Clone X54-5/7.1	1:150	BD Pharmingen	Cat # 558539 RRID: AB_647120
Anti-mouse NK1.1-AF700, Clone PK136	1:100	Invitrogen	Cat # 56-5941-82 RRID: AB_2574505
Anti-mouse MHC Class II (I-A/I-E)-AF700, Clone M5/114.15.2	1:200	Invitrogen	Cat # 56-5321-82 RRID: AB_494009
Anti-mouse MHC Class II (I-A/I-E)-APC-eFluor780, Clone M5/114.15.2	1:200	eBioscience	Cat # 47-5321-82 RRID: AB_1548783
Anti-mouse TCRγδ-BV421, Clone GL3	1:200	BD Horizon	Cat # 562892 RRID: AB_2737871
Anti-mouse Ly6C-BV570, Clone HK1.4	1:600	BioLegend	Cat # 128029 RRID: AB_10896061
Anti-mouse CD11b-BV605, Clone M1/70	1:600	BD Horizon	Cat # 563015 RRID: AB_2737951
Anti-mouse CD11c-BV650, Clone HL3	1:400	BD Horizon	Cat # 564079 RRID: AB_2725779
Anti-mouse CD45.2-BV711, Clone 104	1:400	BD Horizon	Cat #: 563685 RRID: AB_2738374
Anti-mouse CD3ε-BV785, Clone 145-2C11	1:200	BioLegend	Cat # 100355 RRID: AB_2565969
Anti-mouse Siglec-F (CD170)-PECF594, Clone E50-2440	1:800	BD Horizon	Cat # 562757 RRID: AB_2687994
Anti-mouse Ly6G-PECy7, Clone IA8	1:1200	BD Pharmingen	Cat # 560601 RRID: AB_1727562
Anti-mouse CD44-AF700, Clone IM7	1:200	BD Pharmingen	Cat # 560567 RRID: AB_1727480
Anti-mouse CD45.1-APC-Cy7, Clone A20	1:200	BioLegend	Cat # 110715 RRID: AB_313504
Anti-mouse CD45.1-APC, Clone A20	1:200	BioLegend	Cat # 110705 RRID: AB_313494

Anti-mouse TCRVα2-PE-Cy7, Clone B20.1	1:200	BD Pharmingen	Cat # 560624 RRID: AB_1727584
Anti-mouse TCRVα2-FITC, Clone B20.1	1:100	BD Pharmingen	Cat # 561078 RRID: AB_394760
Anti-mouse CD8α-APC-Cy7, Clone 53-6.7	1:200	BD Pharmingen	Cat # 561967 RRID: AB_396769
Anti-mouse NRP1-PECy7, Clone 3E12	1:800	BioLegend	Cat # 145212 RRID: AB_2562360
Anti-mouse IL-2 FITC, Clone JES6-5H4	1:100	BD Pharmingen	Cat # 554427 RRID: AB_395385
Anti-mouse TNF APC, Clone MP6-XT22	1:200	BD Pharmingen	Cat # 561062 RRID: AB_398553
Anti-mouse IFNγ PE-Cy7, Clone XMG1.2	1:200	BD Pharmingen	Cat # 557649 RRID: AB_396766
Anti-mouse TCF1-AF488, Clone S33-966	1:100	BD Pharmingen	Cat # 567018
Anti-mouse Tox-eFluor660, Clone TXRX10	1:200	Invitrogen	Cat # 50-6502-82 RRID: AB_2574265
Anti-mouse CX3CR1-BV711, Clone SA011F11	1:2	BioLegend	Cat # 149031 RRID: AB_2565939
Anti-mouse KLRG1-BV605, Clone 2F1/KLRG1	1:200	BioLegend	Cat # 138419 RRID: AB_2563357
Anti-mouse Eomes-PerCP-eFluor, Clone Dan11Mag	1:200	Invitrogen	Cat # 46-4875-82 RRID: AB_10597455
Anti-mouse Granzyme A-PECy7, Gza-3G85	1:50	Invitrogen	Cat # 25-5831-82 RRID: AB_2573476
Anti-human Granzyme B-AF700, Clone GB11	1:50	BD Pharmingen	Cat # 560213 RRID: AB_1645453
Anti-mouse Perforin-APC, Clone S16009A	1:100	BioLegend	Cat # 154303 RRID: AB_2721462
Anti-mouse CD25-PECF594, Clone PC61	1:200	BD Horizon	Cat # 562694 RRID: AB_2744346

Anti-human CD3-PECF594, Clone UCHT1	1:800	BD Horizon	Cat # 562280 RRID: AB_11153674
Anti-human CD4-BV650, Clone SK3	1:200	BD Horizon	Cat# 563875 RRID: AB_2744425
Anti-human CD8-PerCPCy5.5, Clone SK1	1:200	BD Pharmingen	Cat # 565310 RRID: AB_2687497
Anti-human CD218a/IL18Ra-PE, Clone H44	1:200	BioLegend	Cat # 313808 RRID: AB_345314
Anti-human CD14-APC-H7, Clone MΦP9	1:50	BD Pharmingen	Cat # 560180 RRID: AB_1645464
Anti-human CD16-AF700, Clone 3G8	1:50	BioLegend	Cat # 302026 RRID: AB_2278418
Anti-human CD56-APC, Clone MEM-188	1:50	BioLegend	Cat # 304610 RRID: AB_314452
Anti-human CD19-BV510, Clone SJ25C1	1:200	BD Horizon	Cat #562947 RRID: AB_2737914
Anti-human HLA-DR-BV605, Clone L243	1:50	BioLegend	Cat # 307640 RRID: 2561913
BV421 Streptavidin	N/A	BD Horizon	Cat # 563259 RRID: AB_2869475
PE Streptavidin	N/A	BD Pharmingen	Cat # 554061 RRID: AB_10053328
APC Streptavidin	N/A	BD Pharmingen	Cat # 554067 RRID: AB_10050396
LIVE/DEAD Fixable Aqua Stain	1:500	Invitrogen	Cat # L34957
LIVE/DEAD Fixable Near-IR Dead Cell Stain	1:500	Invitrogen	Cat # L34975
Violet Proliferation Dye 450	N/A	BD Horizon	Cat # 562158

**Influenza virus strains**	
Influenza A Virus: A/HKx31	*Marshall D, et al PNAS, 2001*
Influenza A Virus: A/PR8	*Marshall D, et al PNAS, 2001*
Influenza A Virus: A/HKx31-OVA	*Jenkins et al, J Immunol, 2006*

T cell peptides		
$NP_{366-374}$ (ASNENMETM)	*Townsend et al* GenScript	Custom order
$PA_{224-233}$ (SSLENFRAYV)	*Belz et al* GenScript	Custom order

Commercial kits

LEGENDplex Mouse Anti-Virus Response Panel (13-plex) with Filter Plate	BioLegend	Cat # 740621
RNeasy Plus Micro Kit	Qiagen	Cat # 74004
BD Cytofix/Cytoperm Fixation/Permeabilization Kit	BD Biosciences	Cat # 554714
eBioscience Foxp3 / Transcription Factor Staining Buffer Set	Invitrogen	Cat # 00-5523-00
BD FACS Lysing Solution 10X Concentrate	BD Biosciences	Cat #349202

Mouse strains

Mouse: C57BL/6J		
Mouse: C57BL/6-Tg(TcraTcrb)1100Mjb/J (OT-I)	*Hogquist et al, Cell, 1994*	
