# Supplementary material for: High expression of interleukin-18 receptor alpha correlates with severe respiratory viral disease and defines T cells with reduced cytotoxic signatures
Source: Nat Commun. 2025 Nov 24;16:10344. doi: 10.1038/s41467-025-65262-5 (PMC12644894; doi:10.1038/s41467-025-65262-5)
Supplement: Supplementary file 2 — Reporting Summary [file 41467_2025_65262_MOESM2_ESM.pdf]

nature portfolio

Corresponding author(s): Katherine Kedzierska

Last updated by author(s): 1/10/2025

Reporting Summary

Nature Portfolio wishes to improve the reproducibility of the work that we publish. This form provides structure for consistency and transparency in reporting. For further information on Nature Portfolio policies, see our Editorial Policies and the Editorial Policy Checklist.

Statistics

For all statistical analyses, confirm that the following items are present in the figure legend, table legend, main text, or Methods section.

n/a	Confirmed	
☐	☒	The exact sample size (n) for each experimental group/condition, given as a discrete number and unit of measurement
☐	☒	A statement on whether measurements were taken from distinct samples or whether the same sample was measured repeatedly
☐	☒	The statistical test(s) used AND whether they are one- or two-sided<br>*Only common tests should be described solely by name; describe more complex techniques in the Methods section.*
☐	☒	A description of all covariates tested
☐	☒	A description of any assumptions or corrections, such as tests of normality and adjustment for multiple comparisons
☐	☒	A full description of the statistical parameters including central tendency (e.g. means) or other basic estimates (e.g. regression coefficient) AND variation (e.g. standard deviation) or associated estimates of uncertainty (e.g. confidence intervals)
☐	☒	For null hypothesis testing, the test statistic (e.g. F, t, r) with confidence intervals, effect sizes, degrees of freedom and P value noted<br>*Give P values as exact values whenever suitable.*
☒	☐	For Bayesian analysis, information on the choice of priors and Markov chain Monte Carlo settings
☒	☐	For hierarchical and complex designs, identification of the appropriate level for tests and full reporting of outcomes
☒	☐	Estimates of effect sizes (e.g. Cohen's d, Pearson's r), indicating how they were calculated

Our web collection on statistics for biologists contains articles on many of the points above.

Software and code

Policy information about availability of computer code

Data collection	BD FACS DIVA v8.0.1
Data analysis	FlowJo v10, Prism v10.3.1, RStudio 2022.07.2+576 "Spotted Wakerobin" Release, LEGENDplex™ Data Analysis website (https://legendplex.qognit.com/workflow), bcbio-nextgen v 1.2.8, Samtools 59 v 1.9, Salmon 60 v 1.4.0, STAR 61 v 2.6.1d, FastQC 62 v 0.11.8, MultiQC 63 v1.10.1, edgeR v 3.38.4 in R v 4.2.1, biomaRt 64 v 2.52.0, org.Mm.eg.db 65 v 3.15.0, ggplot2 66 v 3.3.6, EnhancedVolcano 67 v 1.14.0, ComplexHeatmap 68 v 2.12.1, Cytoscape 69 v 3.9.1, stringApp 70 v 1.7.1, clusterMaker2 71 v 2.2

For manuscripts utilizing custom algorithms or software that are central to the research but not yet described in published literature, software must be made available to editors and reviewers. We strongly encourage code deposition in a community repository (e.g. GitHub). See the Nature Portfolio guidelines for submitting code & software for further information.

Data

Policy information about availability of data

All manuscripts must include a data availability statement. This statement should provide the following information, where applicable:

- Accession codes, unique identifiers, or web links for publicly available datasets
- A description of any restrictions on data availability
- For clinical datasets or third party data, please ensure that the statement adheres to our policy

All data generated or analysed during this study are included in this published article (and its supplementary information files) or deposited online. RNAseq data were deposited with the NCBI Sequence Read Archive (#PRJNA1190505). Microarray data from patients infected with A(H7N9) were obtained from GEO accession GSE268303. Bulk RNAseq data from healthy participants, pediatric patients hospitalized with SARS-CoV-2 infection, and pediatric patients hospitalized with MIS-C were obtained from NCBI Sequence Read Archive (SRA) BioProject PRJNA1116218. Single-cell RNAseq data from human tracheal aspirates were obtained from SRA

BioProject PRJNA971535. Microarray data from human respiratory virus challenge studies were obtained from GEO accession GSE73072. Raw FACS data are shown in the manuscript. FACS-source files are available from the authors upon request.

Field-specific reporting

Please select the one below that is the best fit for your research. If you are not sure, read the appropriate sections before making your selection.

- [x] Life sciences
- [ ] Behavioural & social sciences
- [ ] Ecological, evolutionary & environmental sciences

For a reference copy of the document with all sections, see nature.com/documents/nr-reporting-summary-flat.pdf

Life sciences study design

All studies must disclose on these points even when the disclosure is negative.

Sample size	Sample size was >3 as per typical experimental design and mouse availability.
Data exclusions	No data were excluded
Replication	Mouse experiments were performed at least twice.
Randomization	Mice of similar age and same sex were assigned into experimental groups
Blinding	Experiments were not blinded.

Reporting for specific materials, systems and methods

We require information from authors about some types of materials, experimental systems and methods used in many studies. Here, indicate whether each material, system or method listed is relevant to your study. If you are not sure if a list item applies to your research, read the appropriate section before selecting a response.

Materials & experimental systems

n/a	Involved in the study	
[ ]	[x]	Antibodies
[x]	[ ]	Eukaryotic cell lines
[x]	[ ]	Palaeontology and archaeology
[ ]	[x]	Animals and other organisms
[ ]	[x]	Human research participants
[x]	[ ]	Clinical data
[x]	[ ]	Dual use research of concern

Methods

n/a	Involved in the study	
[x]	[ ]	ChIP-seq
[ ]	[x]	Flow cytometry
[x]	[ ]	MRI-based neuroimaging

Antibodies

Antibodies used	We used commercially-available antibodies as per Material and Methods.
Validation	Each antibody used had a validated technical data sheet as per manufacturer's website showing positive staining, and titrated in our laboratory prior to their use. FACS positive staining is shown in the FACS plots in the main figures and extended data figures.

Animals and other organisms

Policy information about studies involving animals; ARRIVE guidelines recommended for reporting animal research

Laboratory animals	C57BL/6,olah-/- and OT-1 strains of mice, 7-12 weeks of age and of both sexes were used. Mice were bred and maintained at the Melbourne Bioresources Platform facilities (University of Melbourne) under a 12h/12h light/dark cycle, at 19-22°C and 40-70% humidity. This information is in the Materials and Methods.
Wild animals	No wild animals were used
Field-collected samples	No field-collected samples were used
Ethics oversight	All animal work was conducted in accordance with the Australian National Health and Medical Research Council (NHMRC) Code of Practice for the Care and Use of Animals and approved by the Animal Ethics Experimentation Committee (AEC 20296) at the University of Melbourne.

Note that full information on the approval of the study protocol must also be provided in the manuscript.

Human research participants

Policy information about studies involving human research participants

Population characteristics	Patients infected with A(H7N9) influenza virus were admitted to the Shanghai Public Health Clinical Center (SHAPHC), their clinical details, and age and gender were previously published 2,13.<br>COVID-19 pediatric cohort 15 consisted of participants aged 0-21 years and was obtained as part of the Overcoming COVID-19 Study under IRB at Boston Children's Hospital (IRB-P00033157).<br>RSV infected children aged 0-2 were recruited at LeBonheur Children's Hospital (Memphis, TN, USA) 21 .<br>Healthy individuals (n=17) were 82% female and aged between 19-52 (median 22). They were recruited under the University of Melbourne Human Research Ethics Committee (#13344 and #29132).<br>Hospitalized patients with acute respiratory infections consisted of 51 participants, 51% were female and cohort was aged between 26-89 (median 68). This cohort was recruited under the Monash Health (HREC/15/MonH/64), Austin Health (SSA/28204/Austin-2022) and the Alfred Hospital (#280/14) Human Research Ethics Committees.
Recruitment	A(H7N9) patients were admitted to the Shanghai Public Health Clinical Center, as per our previous studies 2, 13. Informed consent was obtained from participants, and the study was approved and conducted under supervision by the SHAPHC Ethics Committee. Blood RNAseq data from the COVID-19 pediatric cohort was obtained from hospitalized patients aged 0-21 years as part of the Overcoming COVID-19 Study under IRB at Boston Children's Hospital (IRB-P00033157). Blood samples were collected early after admission. Tracheal aspirate samples were obtained from RSV infected children aged 0-2 recruited at LeBonheur Children's Hospital (Memphis, TN, USA) 21. IL18R1 expression was also analysed across time for human challenge models of mild respiratory infections (H1N1 DEE3 n=477, H3N2 DEE2 n=355, HRV Duke n=471, RSV DEE4 n=420), using previously published datasets 22. Healthy individuals were recruited via the University of Melbourne. Hospitalized patients with acute respiratory infections were recruited from Austin Health and the Alfred Hospital. All participants provided informed written consent.
Ethics oversight	Experiments conformed to the Declaration of Helsinki Principles and the Australian National Health and Medical Research Council Code of Practice. Ethics approval was provided by the Human Research Ethics Committee of the University of Melbourne (#13344 and #29132), Monash Health (HREC/15/MonH/64), Austin Health (SSA/28204/Austin-2022) and the Alfred Hospital (#280/14). Study conducted in Shanghai Public Health Clinical Center was approved by the SHAPHC Ethics Committee. Data from COVID-19 pediatric cohort from the Overcoming COVID-19 Study was obtained under IRB at Boston Children's Hospital (IRB-P00033157). Data from tracheal aspirate samples from RSV infected children were obtained under IRB at the University of Tennessee (17-05744-XP STJUDE) and St. Jude Children's Research Hospital (IRB Pro00008351).

Note that full information on the approval of the study protocol must also be provided in the manuscript.

Flow Cytometry

Plots

Confirm that:

- [x] The axis labels state the marker and fluorochrome used (e.g. CD4-FITC).
- [x] The axis scales are clearly visible. Include numbers along axes only for bottom left plot of group (a 'group' is an analysis of identical markers).
- [x] All plots are contour plots with outliers or pseudocolor plots.
- [x] A numerical value for number of cells or percentage (with statistics) is provided.

Methodology

Sample preparation	Samples were prepared as described in Methods
Instrument	BD FACSAriaIII was used for acquisition of data
Software	BD FACS Diva v8.0.1, FlowJo v10
Cell population abundance	Frequencies are shown within each result dot plot in the main figures
Gating strategy	Gating strategies have been presented in the Supplementary Figures

- [x] Tick this box to confirm that a figure exemplifying the gating strategy is provided in the Supplementary Information.
